# Pericardial Cyst at the Left Cardiophrenic Angle Mimicking Localized Effusion: A Diagnostic Dilemma

**DOI:** 10.7759/cureus.103985

**Published:** 2026-02-20

**Authors:** André Veloso, Sofia Néri Fialho, Cláudia Guerreiro, Rute Martins, Vanda Areias

**Affiliations:** 1 Department of Pulmonology, Unidade Local de Saúde Algarve - Hospital de Faro, Faro, PRT; 2 Department of Radiology, Unidade Local de Saúde Algarve - Hospital de Faro, Faro, PRT; 3 Faculty of Medicine and Biomedical Sciences, Universidade do Algarve, Faro, PRT

**Keywords:** benign cystic tumor, middle mediastinal mass, pericardial cyst, thoracic mri, transesophageal echocardiography

## Abstract

Pericardial cysts are rare, benign, congenital anomalies of the pericardium, usually located within the right cardiophrenic space, often discovered incidentally during imaging performed for unrelated reasons. They usually do not cause symptoms and are detected by chance. Chest X-ray, echocardiography, and chest computed tomography (CT) or magnetic resonance imaging (MRI) are useful diagnostic tools. Nevertheless, after safe diagnostic, management options include conservative management with follow-up, percutaneous aspiration of the cyst, and excision of the cyst (direct invasive therapeutic approach should be the option only for patients with overt symptoms and indications). We report a case of a large, stable pericardial cyst in an atypical location in a 64-year-old woman.

## Introduction

Pericardial cysts are benign mediastinal lesions that are most frequently discovered incidentally due to their infrequent clinical presentation. These lesions are most commonly identified in individuals in their third or fourth decade of life, with no sex predilection. Pericardial cysts are usually congenital in origin, but other causes of origin of pericardial cysts have also been described in the literature [1,2]. They constitute approximately 7% of mediastinal masses, 33% of mediastinal cysts, and about 5% of all thoracic cysts [1,2]. The majority of pericardial cysts are located at the right cardiophrenic angle (70%), followed by the left cardiophrenic angle (22%), with the remaining 8% found in atypical anterior-superior or posterior mediastinal locations [1,3].

Between 50% and 75% of pericardial cysts are asymptomatic and are detected during imaging performed for unrelated indications [1,3]. When symptoms are present, they typically result from compression of adjacent structures such as the heart, great vessels, esophagus, or tracheobronchial tree, and may present as chronic cough, chest pain, dyspnea, or retrosternal pressure [1,3,4]. Less common manifestations include palpitations secondary to arrhythmias and recurrent lower respiratory tract infections [1,2]. Rarely, presentations such as syncope, pneumonia, congestive heart failure, and sudden cardiac death have been reported [1,3,4].

Computed tomography (CT) is the preferred diagnostic modality, as it provides detailed visualization of pericardial anatomy and enables precise localization and characterization of pericardial lesions, thereby guiding management [2,5,6]. Diagnostic difficulties may occur when cysts are located in atypical sites or when the cyst fluid contains elevated protein levels. Magnetic resonance imaging (MRI) is also valuable, with pericardial cyst fluid typically appearing hyperintense on T2-weighted images and hypointense on T1-weighted images. However, increased protein content can alter these signal characteristics, complicating differentiation from hematomas or neoplasms [1,2,5,6]. Diffusion-weighted imaging may be beneficial in selected cases [1,2,5,6]. Echocardiography is important for functional assessment and longitudinal follow-up, but is less reliable as a primary diagnostic tool due to limited acoustic windows and challenges in identifying cysts in atypical locations [1-3,5,6].

Although generally benign, pericardial cysts may lead to complications such as inflammation, hemorrhage, rupture, or compression of right-sided cardiac chambers, potentially resulting in elevated venous pressures, peripheral venous congestion, ascites, hepatomegaly, and right-sided heart failure [1,4]. Management options include conservative observation, percutaneous aspiration, and surgical excision. Surgical intervention is recommended for symptomatic patients, large cysts, radiologic evidence of compression or impending compression, or concern for malignant transformation [1,4]. Surgery may also prevent life-threatening complications such as cardiac tamponade, bronchial obstruction, or sudden cardiac death [1,4]. Asymptomatic patients may be managed conservatively with serial echocardiographic monitoring for functional assessment or thoracic CT/MRI to evaluate growth, compressive effects, hemorrhage, infection, or rupture [1,4,6].

## Case presentation

**Figure 1:**
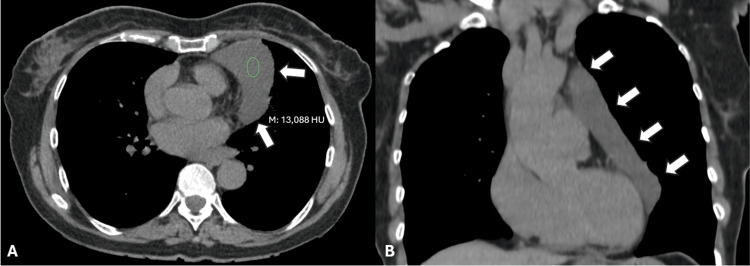
(A) Soft tissue window axial non-contrast chest CT revealing a large, well-circumscribed, homogeneous lesion with fluid attenuation (medium density of 13 HU), adjacent to the pericardium (white arrows). (B) Soft tissue window coronal non-contrast chest CT showing the fluid attenuation lesion, with extension to the aortic arch (white arrows). Fat planes with the heart chambers and aortic arch can be seen. CT: computed tomography, HU: Hounsfield unit

We report the case of a 64-year-old woman, a non-smoker, but with passive exposure to tobacco (through her husband), working as a supermarket cashier. Her past medical history includes dyslipidemia and a prior SARS-CoV-2 infection, associated with chronic cough. Current medication includes rosuvastatin/ezetimibe 20/10 mg, fluticasone furoate/vilanterol 92/22 µg, and trazodone 150 mg. She denies known drug allergies and beginning of new medication. Family history is notable for heart failure in her mother, gastric and colon cancer in her father, and pulmonary disease of unspecified nature in one sibling. The patient was referred to a pulmonology consultation with a reported progressive fatigue for approximately one year duration and concomitant productive cough with mucoid sputum, predominantly in the morning. Due to this clinical presentation, her family doctor, before pulmonology consultation, ordered an echocardiogram that suggested a possible pericardial effusion. A subsequent chest CT revealed a large, thin-walled cystic lesion along the left lateral cardiac wall, measuring 13.7 × 7.4 × 5.5 cm, suggestive of a pericardial cyst, less probable a thymic cyst (Figure 1).

**Figure 2:**
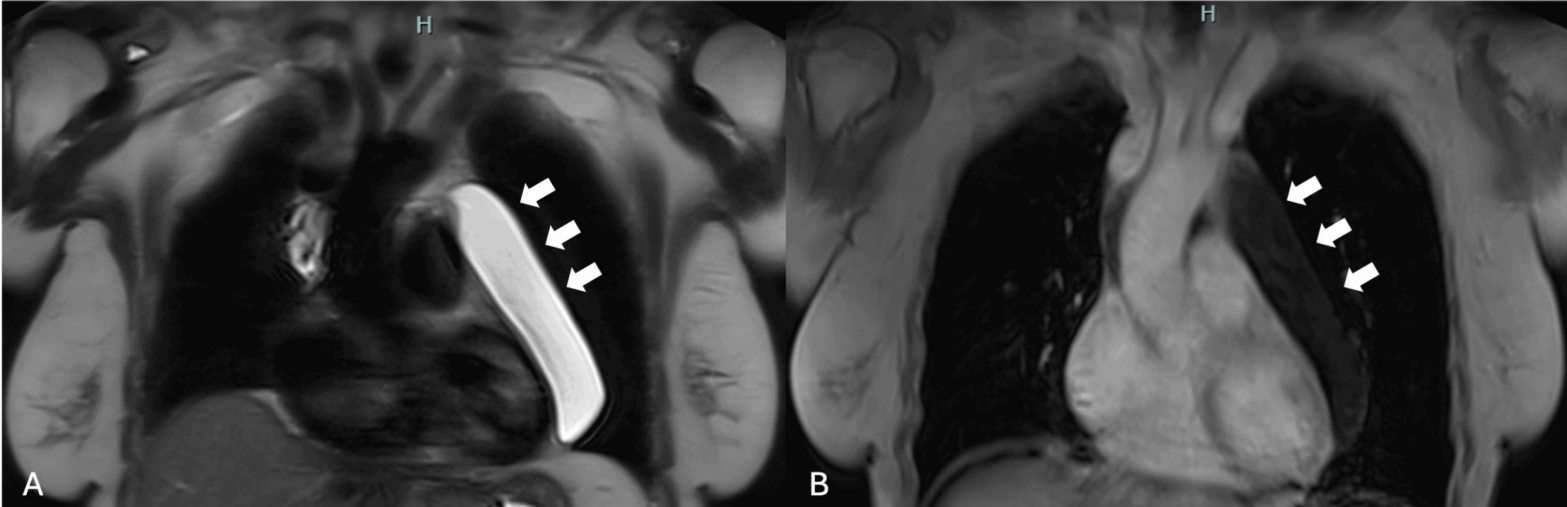
(A) Coronal T2-weighted MR image shows a well-defined lesion (white arrows), with thin walls and homogenous high signal intensity. (B) Coronal T1-weighted MR image shows the lesion with low signal intensity (white arrows). MR: magnetic resonance

**Figure 3:**
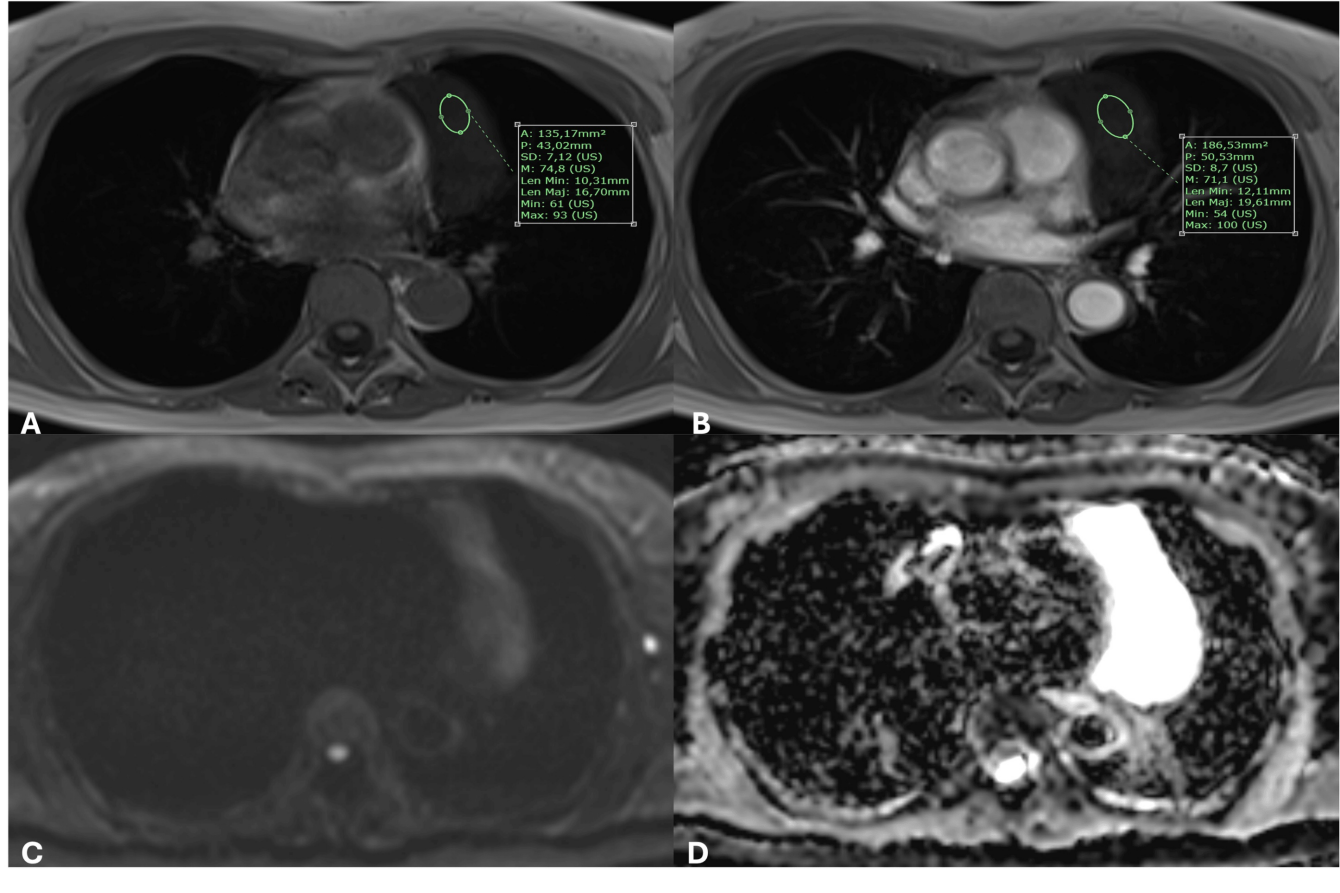
(A) T1-weighted sequences with intravenous contrast administration (gadolinium) do not reveal any enhancement. (B-D) DWI and ADC map show no signs of restriction to diffusion within the lesion. Overall, no mass effect, invasion of adjacent structures, or pericardiac effusion is noted. DWI: diffusion-weighted imaging, ADC: apparent diffusion coefficient

In consultation, no changes in physical examination or vital signs were observed. A pulmonary function test was ordered, showing a mild restrictive defect (total lung capacity (TLC): 3.79l%-78%, z score: -1.88). Thoracic MRI confirmed a well-defined, homogeneous, T2-hyperintense and T1-hypointense lesion with no enhancement after contrast, consistent with a fluid-filled cyst. The lesion measured approximately 12 × 3 × 8.4 cm, extending from the level of the aortic arch to the left hemidiaphragm, encasing the left cardiac contour without evidence of mass effect, invasion, or pericardial effusion, suggestive of a pericardial cyst (Figure 2 and Figure 3). Differential diagnoses, including cystic lymphangioma, were considered less likely due to the homogeneous content and absence of septations or enhancement.

**Figure 4:**
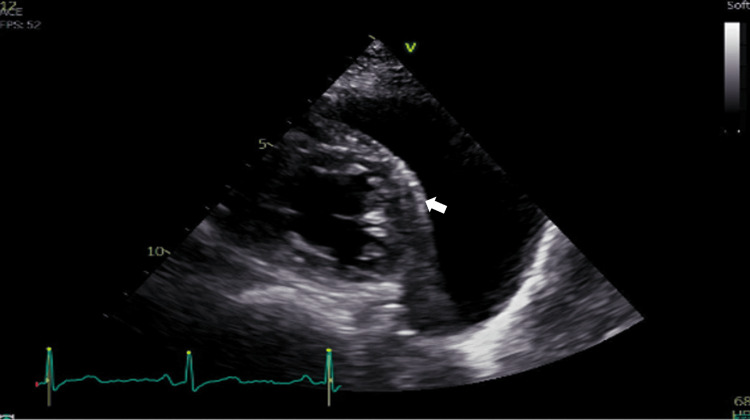
Transthoracic echocardiogram showing a moderate-sized liquid collection located along the left side wall of the heart (arrow) with possible diagnosis between pericardial effusion or pericardial cyst (echocardiogram limited for extracardiac lesion characterization). This examination confirmed normal biventricular dimensions and function, with no hemodynamic compression by the collection.

A follow-up transthoracic echocardiogram confirmed normal biventricular dimensions and function (preserved LV and RV systolic function, no significant valvular disease, and low probability of pulmonary hypertension). The previously described large pericardial cyst was visualized, without hemodynamic compression (Figure 4).

**Figure 5:**
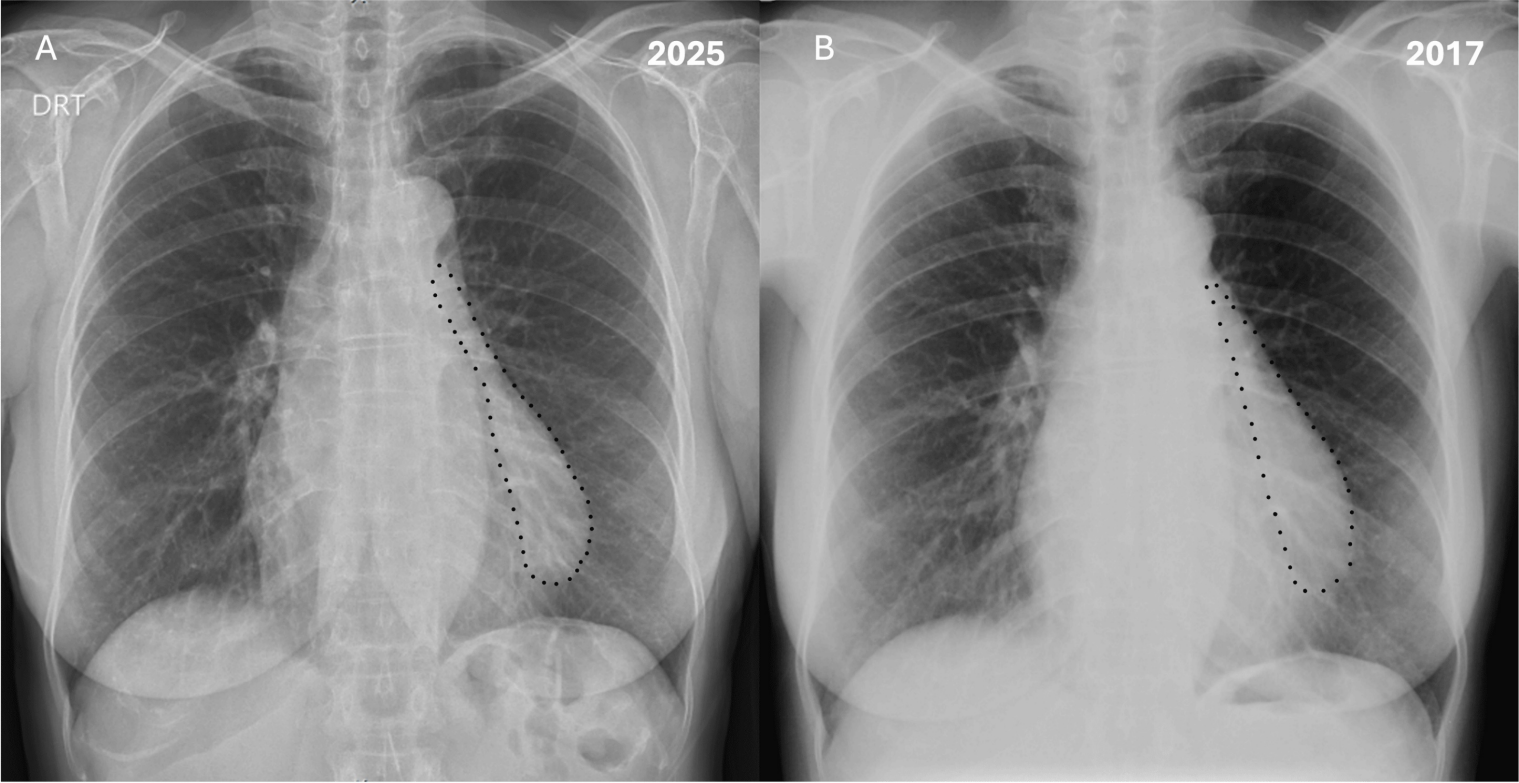
(A) PA chest X-ray showing an enlargement of the upper left heart border, a smooth, well-defined convex opacity, sharply marginated, rounded teardrop-shaped, with clear lung parenchyma. A mass (black dots) is hardly depicted. (B) Retrospectively, in a PA chest X-ray taken eight years earlier, it is evident that the opacity in the left cardiac border, marked by a dotted line, was already present, which reveals no significant interval change in the appearance, size, or location, morphologically and chronologically consistent with a possible benign, stable pericardial cyst. PA: posteroanterior

After discussion with radiology consultant, a retrospective review of a previous chest radiograph showed that the cystic image was already present and appears radiographically stable over eight years (Figure 5).

Given the stability and benign imaging features, positron emission tomography (PET)-CT was deemed unnecessary, and a repeat thoracic MRI in one year was recommended for follow-up.

## Discussion

A pericardial cyst is a rare condition with an incidence of one in 100,000 [1,2,5,6]. They are usually unilocular and contain a clear, water-like fluid. The cysts can vary in size, ranging from 2 to 28 cm [1,2,5,6]. Pericardial cysts are usually asymptomatic, but in some cases, symptoms may occur because of compression of adjacent structures. In symptomatic cases, patients may present with chest pain, cough, fever, and arrhythmias [1,3,4]. Rarely, they may result in serious complications such as erosion into vascular structures, pericarditis, obstruction of the right ventricular outflow tract, pulmonary stenosis, or sudden death [1,3,4]. Pericardial cysts are uncommon benign lesions most frequently located in the right cardiophrenic angle, whereas left-sided lesions represent only about 22% of all pericardial cysts, making the location in our patient distinctly unusual [1,2,5,6]. This atypical topography can complicate diagnostic interpretation, as cysts outside the typical cardiophrenic recess may mimic other mediastinal or paracardiac masses, including thymic, bronchogenic, enteric, or lymphangiomatous cysts [1,5]. The combination of a left-sided position and large dimensions in the present case highlights the importance of careful radiologic evaluation to avoid misclassification and unnecessary invasive procedures.

A key feature of this case is the documented long-term radiographic stability, with retrospective imaging confirming the lesion’s presence and unchanged characteristics for at least eight years. Stability over time is strongly associated with benign behavior and greatly reduces the likelihood of malignancy or aggressive pathology. This aligns with published data demonstrating that most pericardial cysts remain stable or grow slowly, if at all, and rarely undergo complications such as hemorrhage, rupture, or infection [1,5].

Echocardiography is often the first-line modality. Color Doppler imaging assists in confirming the fluid-filled nature of the lesion and excludes vascular differentials such as left ventricular aneurysm, aortic aneurysm, or a prominent left atrial appendage [1,5]. Misinterpretation of a pericardial cyst as pericardial effusion on initial transthoracic echocardiography is a well-recognized diagnostic pitfall. Because echocardiography provides a limited field of view and relies on acoustic windows, extracardiac cystic structures, particularly those located laterally or adjacent to the cardiac contour, may appear as anechoic spaces surrounding the heart, mimicking fluid within the pericardial sac [1,5]. This is especially true for large cysts with broad pericardial contact, as seen in our patient. Two-dimensional echocardiography lacks the spatial resolution to reliably distinguish between fluid within the pericardial cavity and a well-defined paracardiac lesion [1,5]. For this reason, echocardiography is useful for functional assessment (in this case, it established the absence of hemodynamic compression) but is not the optimal primary diagnostic modality, as atypically located lesions are often missed or misinterpreted [1,2,5]. The subsequent CT and MRI in this case were essential to correctly identify the structure as a pericardial cyst rather than a true pericardial effusion [1-3].

Chest CT offers excellent anatomical definition and typically shows a well-circumscribed, homogeneous, fluid-attenuation lesion adjacent to the pericardium [1,3,5]. Although pericardial cysts are historically considered congenital, communication with the pericardial sac is rarely demonstrated even at surgery. Variations in CT density may occur when the cystic content is proteinaceous or hemorrhagic, although our patient’s lesion showed classic fluid attenuation without septations or enhancement [1,3,5]. MRI further supported a benign diagnosis, demonstrating low-to-intermediate signal on T1-weighted images and high signal on T2-weighted sequences without contrast enhancement, features characteristic of simple cystic structures [1,5]. This diagnostic consistency across modalities obviated the need for biopsy or PET-CT.

Regarding pathogenesis, pericardial cysts are typically believed to be congenital, arising from incomplete coalescence of mesenchymal lacunae forming the pericardial sac [1,4,5]. Acquired forms, although less common, have been associated with prior cardiac surgery, trauma, infections (such as tuberculosis or echinococcosis), pericarditis, metastasis, or long-term hemodialysis [1,4,5]. In this patient, the absence of prior cardiac interventions, trauma, infectious history, or systemic inflammatory disease, combined with stable imaging features and the cyst’s simple morphology, strongly supports a congenital origin. Despite measuring approximately 12 cm, placing it at the upper range of reported pericardial cyst dimensions [1,4,5], the lesion produced no mass effect on echocardiography or MRI and no hemodynamic compromise. The literature does not define a strict size threshold mandating surgical intervention; instead, management is typically guided by symptoms, radiologic suspicion, or compression of adjacent structures. Even large cysts can remain clinically silent, as in this case. The patient’s mild restrictive pattern on pulmonary function testing may be attributable to the cyst’s anatomical proximity to the left hemidiaphragm and left lung, although the absence of demonstrable compressive imaging findings makes a causal relationship uncertain. Given the stability of both the lesion and the respiratory symptoms, conservative monitoring was preferred. Long-term clinical monitoring is planned for this patient, with serial echocardiographic evaluations to monitor for potential expansion or the development of compressive symptoms.

Another relevant aspect is the decision to avoid biopsy or surgical excision. In pericardial cysts, tissue sampling rarely contributes diagnostic value, as imaging typically provides a high degree of specificity [1,5]. Moreover, invasive procedures carry risks, including infection, hemorrhage, and potential injury to cardiac or mediastinal structures [1,5]. Simple drainage is generally not recommended, as these cysts typically recur without complete resection [1,5,6]. Surgical excision is generally reserved for symptomatic patients, cysts with suspicious imaging features, rapidly enlarging lesions, or those associated with compression of vital structures [1,5,6]. None of these criteria were met in the present case. Multimodal imaging, including CT, MRI, and echocardiography, consistently demonstrated a homogeneous, thin-walled, non-enhancing cyst without signs of invasion, supporting a benign etiology [1,5]. The decision for conservative management with periodic imaging follows current recommendations for asymptomatic or minimally symptomatic patients with uncomplicated pericardial cysts [1,5,6]. MRI was selected as the primary modality for follow-up due to its superior tissue characterization and lack of ionizing radiation. PET-CT was appropriately deemed unnecessary, as metabolic evaluation is not typically indicated in lesions with unequivocal benign morphology.

## Conclusions

This case illustrates an incidentally discovered middle mediastinal cystic lesion, likely of pericardial origin, that has remained radiographically stable for several years. Long-term stability, careful correlation of imaging findings, and benign imaging features can reliably guide clinicians toward a conservative approach, avoiding unnecessary invasive diagnostic interventions as was in this clinical case. Due to the inherent challenges in differentiating pericardial cysts from localized effusions via transthoracic echocardiography, the use of CT and MRI is essential for tissue characterization and diagnostic clarity. Once the diagnosis is established, echocardiography serves as the primary modality for long-term surveillance, specifically to monitor for cyst expansion or the emergence of new symptoms. This multi-modality approach ensures an accurate initial diagnosis while providing a cost-effective and non-invasive means of clinical follow-up. Future management of this patient will consist of regular clinical surveillance and repeat echocardiography to evaluate for any morphological changes or symptomatic progression. This case highlights the importance of imaging in differentiating pericardial cysts from other mediastinal masses, underscores their typically benign course, and contributes to the growing body of evidence supporting surveillance over surgery in stable, asymptomatic, or minimally symptomatic pericardial cysts, even when they are unusually large or left-sided.
